# Increased Likelihood of Detecting Ebola Virus RNA in Semen by Using Sample Pelleting

**DOI:** 10.3201/eid2704.204175

**Published:** 2021-04

**Authors:** Courtney M. Bozman, Mosoka Fallah, Michael C. Sneller, Catherine Freeman, Lawrence S. Fakoli, Bode I. Shobayo, Bonnie Dighero-Kemp, Cavan S. Reilly, Jens H. Kuhn, Fatorma Bolay, Elizabeth Higgs, Lisa E. Hensley

**Affiliations:** National Institutes of Health, Frederick, Maryland, USA (C.M. Bozman, B. Dighero-Kemp, J.H. Kuhn, L.E. Hensley);; National Public Health Institute of Liberia, Monrovia, Liberia (M. Fallah, C. Freeman, L.S. Fakoli III, B.I. Shobayo, F. Bolay);; National Institutes of Health, Bethesda, Maryland, USA (M.C. Sneller, E. Higgs);; University of Minnesota, Minneapolis, Minnesota, USA (C.S. Reilly)

**Keywords:** Ebola virus, EBOV, Ebola virus disease, EVD, ebolavirus, viruses, virus RNA, sample pelleting, persistence, semen, sexual transmission, zoonoses, Liberia

## Abstract

Ebola virus RNA can reside for months or years in semen of survivors of Ebola virus disease and is probably associated with increased risk for cryptic sexual transmission of the virus. A modified protocol resulted in increased detection of Ebola virus RNA in semen and improved disease surveillance.

During 2013–2016, Ebola virus (EBOV; family *Filoviridae*, genus *Ebolavirus*, species *Zaire ebolavirus*) caused an unprecedented outbreak of Ebola virus disease (EVD) that began in Guinea and subsequently affected Liberia, Sierra Leone, and, to a much lesser degree, several other countries in West Africa. Due in part to the lack of medical infrastructure and response preparedness in these countries, the outbreak ultimately involved 28,652 human infections and 11,325 deaths (*1*,*2*).

The large number of EVD survivors enabled detailed studies, such as the Partnership for Research on Ebola Virus (PREVAIL) III study (*3*), which aimed at characterizing potential EVD sequelae and EBOV persistence in a cohort of 1,144 EVD survivors in Liberia over the course of 5 years. An unexpected observation of these studies was the persistence of EBOV RNA and sometimes-replicating EBOV in the brain, eyes, and semen of survivors (*4*). EBOV RNA persistence in semen of EVD survivors, measurable up to 40 months (*3**,**5*), has been associated with rare events of sexual EBOV transmission and EVD outbreak flareups (*6*).

Assuming a causal relationship between EBOV RNA and EBOV presence in semen, we collaborated with the overseas response team to initiate an ongoing (and unpublished) trial, PREVAIL IV, to counter sexual EBOV transmission from survivors through reduction of viral RNA concentrations in semen by using the candidate medical countermeasure remdesivir. However, interpretation of data obtained in studies such as PREVAIL IV is crucially dependent on the sensitivity of EBOV RNA detection in semen samples.

The GeneXpert Systems (Cepheid, https://www.cepheid.com) are diagnostic platforms that implement single-use cartridges to simultaneously extract and detect RNA by using reverse transcription PCR. During PREVAIL III (*3*), the GeneXpert IV System was applied to standard processing of EBOV survivor semen samples using EBOV nucleoprotein and glycoprotein RNA-specific GeneXpert cartridges Cepheid) (*7*): 100 μL of semen sample was transferred directly into 2.5 mL of lysis buffer provided in the kit and incubated for 10 min, followed by a second incubation of 5 min in presence of 100 μL of 1 M dithiothreitol (Sigma-Aldrich, https://www.sigmaaldrich.com). Within 30 min of processing, 1 mL of this solution was then loaded into the cartridge, run according to the manufacturer’s instructions, and analyzed with GeneXpert Diagnostic Software (*8*).

We sought to further increase the EBOV RNA detection sensitivity (then 3.1% and 2.9% with whole samples) of this protocol for semen. For this experiment, 1,661 EVD survivor samples and nonsurvivor controls from PREVAIL III and IV with sample volumes >1.6 mL were divided into 2 cohorts and processed either as described above (whole sample) or first pelleted (pellet sample) by using 2 replicate experiments each (A and B).

For pelleting, we centrifuged 300 μL of each semen sample at 10,000 × *g* for 10 min. After pelleting, we discarded supernatants, resuspended pellets by pipetting in 100 μL of kit-provided lysis buffer and incubated for 10 min, and incubated for 5 min in presence of 100 μL of 1 M dithiothreitol. Then, we loaded 100 μL of each sample onto cartridges and processed the same way as the standard, unpelleted control sample. Samples were considered valid and positive when both the kit-provided sample processing control and probe check control passed kit criteria and EBOV nucleoprotein or glycoprotein RNA was detected.

Overall, an average of 3.0% of the whole sample-cohort was positive, compared with an average of 5.0% of the pellet-sample cohort, thereby almost doubling the detection rate (p<0.0001) (Table). We observed variability among replicates A and B (0.7% for the pellet and 0.2% for the whole sample), but this difference was not significant (p = 0.35) according to the F-test for the equality of 2 variances.

**Table:** Number of positive samples detected in whole semen samples versus pelleted semen samples for detection of Ebola virus RNA*

Method: replicate	No. samples	No. invalid samples	No. (%) Ebola virus RNA positive samples
Whole sample: A	1,661	66	50 (3.1)
Whole sample: B	1,661	112	45 (2.9)
Pellet sample: A	1,661	85	84 (5.3)
Pellet sample: B	1,661	69	73 (4.6)

*Also included are no. invalid samples (i.e., those that did not pass 1 or both of the controls (sample processing control and probe check control).

Mixed-effects logistic regression models appropriate to the study design (with random effects for specimens and random effects for replicates nested within specimens) yielded an estimated relative sensitivity of 2.24 (95% CI 1.51–2.98; p<0.0001) in favor of the pellet-based procedure. Thus, when semen sample volumes from EVD survivors are >300 μL, we recommend pelleting 300 μL to increase the EBOV RNA detection rate and using the GeneXpert IV System.

## References

[1] Decroo T, Fitzpatrick G, Amone J. What was the effect of the West African Ebola outbreak on health programme performance, and did programmes recover? Public Health Action. 2017;7(Suppl 1):S1–2. 10.5588/pha.17.0029 28744431 PMC5515555

[2] Bullard SG. A day-by-day chronicle of the 2013–2016 Ebola outbreak. Springer: Cham (Switzerland); 2018 [cited 2021 Jan 1]. https://link.springer.com/chapter/10.1007/978-3-319-76565-5_8 10.1080/13623699.2018.1505366 30074403

[3] Sneller MC, Reilly C, Badio M, Bishop RJ, Eghrari AO, Moses SJ, et al.; PREVAIL III Study Group. A longitudinal study of Ebola sequelae in Liberia. N Engl J Med. 2019;380:924–34. 10.1056/NEJMoa1805435 30855742 PMC6478393

[4] Jacob ST, Crozier I, Fischer WA II, Hewlett A, Kraft CS, Vega MA, et al. Ebola virus disease. Nat Rev Dis Primers. 2020;6:13. 10.1038/s41572-020-0147-3 32080199 PMC7223853

[5] Keita AK, Vidal N, Toure A, Diallo MSK, Magassouba N, Baize S, et al.; PostEbogui Study Group. PostEbogui Study Group. A 40-month follow-up of Ebola virus disease survivors in Guinea (PostEbogui) reveals long-term detection of Ebola viral ribonucleic acid in semen and breast milk. Open Forum Infect Dis. 2019;6:ofz482. 10.1093/ofid/ofz482 32128327 PMC7047953

[6] Schindell BG, Webb AL, Kindrachuk J. Persistence and sexual transmission of filoviruses. Viruses. 2018;10:683. 10.3390/v10120683 30513823 PMC6316729

[7] Loftis AJ, Quellie S, Chason K, Sumo E, Toukolon M, Otieno Y, et al. Validation of the Cepheid GeneXpert for detecting Ebola virus in semen. J Infect Dis. 2017;215:344–50. 27932614 10.1093/infdis/jiw562 PMC5965086

[8] Pettitt J, Higgs E, Fallah M, Nason M, Stavale E, Marchand J, et al. Assessment and optimization of the GeneXpert diagnostic platform for detection of Ebola virus RNA in seminal fluid. J Infect Dis. 2017;215:547–53. 28003349 10.1093/infdis/jiw599 PMC6075475

